# Antagonistic experimental coevolution with a parasite increases host recombination frequency

**DOI:** 10.1186/1471-2148-12-18

**Published:** 2012-02-13

**Authors:** Niels AG Kerstes, Camillo Bérénos, Paul Schmid-Hempel, K Mathias Wegner

**Affiliations:** 1 ETH Zürich, Institute of Integrative Biology, Experimental Ecology, CH-8092 Zürich, Switzerland; 2 University of Edinburgh, Institute of Evolutionary Biology, Edinburgh EH9 3JT, UK; 3 Leibniz Institute for Marine Sciences (IfM-Geomar), Evolutionary Ecology of Marine Fishes, Kiel, Germany; 4 Wadden Sea Station Sylt, Alfred Wegener Institute for Polar and Marine Sciences, List/Sylt, Germany

## Abstract

**Background:**

One of the big remaining challenges in evolutionary biology is to understand the evolution and maintenance of meiotic recombination. As recombination breaks down successful genotypes, it should be selected for only under very limited conditions. Yet, recombination is very common and phylogenetically widespread. The Red Queen Hypothesis is one of the most prominent hypotheses for the adaptive value of recombination and sexual reproduction. The Red Queen Hypothesis predicts an advantage of recombination for hosts that are coevolving with their parasites. We tested predictions of the hypothesis with experimental coevolution using the red flour beetle, *Tribolium castaneum*, and its microsporidian parasite, *Nosema whitei*.

**Results:**

By measuring recombination directly in the individuals under selection, we found that recombination in the host population was increased after 11 generations of coevolution. Detailed insights into genotypic and phenotypic changes occurring during the coevolution experiment furthermore helped us to reconstruct the coevolutionary dynamics that were associated with this increase in recombination frequency. As coevolved lines maintained higher genetic diversity than control lines, and because there was no evidence for heterozygote advantage or for a plastic response of recombination to infection, the observed increase in recombination most likely represented an adaptive host response under Red Queen dynamics.

**Conclusions:**

This study provides direct, experimental evidence for an increase in recombination frequency under host-parasite coevolution in an obligatory outcrossing species. Combined with earlier results, the Red Queen process is the most likely explanation for this observation.

## Background

Meiotic recombination breaks down genotypes that have proven to be successful, and therefore it was thought to evolve only under very limited conditions [1]. One hypothesis for the adaptive value of recombination, the Red Queen Hypothesis [2-4], suggests that parasites play an important role in maintaining non-zero recombination rates in their hosts. Parasites are ubiquitous, and parasitism is considered to be one of the most significant selection factors for any organism [5]. According to the Red Queen Hypothesis, hosts and parasites engage in sustained and fluctuating antagonistic coevolution, during which the parasite population continuously adapts to the most common genotypes in the host population [2,3]. Rare host genotypes, by contrast, enjoy a selective advantage and thus will rise in frequency and become common in turn. The parasite population will continue to adapt to host genotypes that are common, but formerly rare, and thus fuel the sustained, fluctuating coevolutionary process. Because rare resistance genotypes are expected to always have a selective advantage under negative frequency-dependent selection, host genotypes will fluctuate over time and genetic diversity is maintained [6,7]. In sexual organisms, rare genotypes are continuously created by meiotic recombination, either via segregation or chromosomal crossovers. As rare recombinants enjoy a selective advantage, recombination should lead to an increase in the mean fitness of host offspring [8] and thus favour the spread of a (linked) recombination modifier in the population. Although theoretical analyses show that the detailed evolutionary processes that favour such modifiers are somewhat more complex [9,10], the essential expectation of the Red Queen Hypothesis still remains that recombination should be selectively favoured under antagonistic host-parasite coevolution.

Mathematical models provide theoretical support for the Red Queen Hypothesis [6,8], even though there is controversy over the general applicability of the hypothesis [11,12]. So far, several empirical studies have tested key assumptions and predictions of the Red Queen, or have analysed whether observational data is compatible with the hypothesis [9]. For example, there is evidence for the predictions that parasites maintain genetic variation in their host populations [13,14], that parasites track the most common host genotypes [15-18], and that parasite infectivity for certain host genotypes fluctuates over time [19]. By contrast, the evolution and maintenance of recombination rate is rarely directly addressed, as most tests deal with the question of what favours sexual over asexual reproduction [20,21], without considering how parasitism may change recombination rate within a sexual host population itself. Several studies give circumstantial evidence for the Red Queen Hypothesis by demonstrating an association of parasitism with genotypic variation within a host population. Such an association has been found in a range of hosts with different reproductive modes. For example, host recombination correlated positively with parasite load in the grasshopper *Eyprepocnemic plorans *[22], and immune genes that directly interact with pathogens cluster in regions of higher recombination frequencies in *Drosophila melanogaster *[23]. Populations of *Caenorhabditis elegans *exposed to *Bacillus thuringiensis *showed changes, such as increased genetic diversity, compatible with the Red Queen Hypothesis [13]. Pathogen-induced stress also directly increased somatic recombination in *Arabidopsis*, which could actually lead to more variation in the progeny as well, since plants lack a predetermined germ line [24]. Similarly, genotypic diversification of offspring by multiple mating led to higher fitness for the mother in *Bombus terrestris *in the face of parasitism in the field [25]. The most significant field study to date involving antagonistic coevolution of the freshwater snail, *Potamopyrgus antipodarium*, a species that has both sexual and asexual populations, with its trematode parasites, shows that sexual (i.e. recombining) snail variants occur more often in environments with high parasite pressure [26,27]. In a recent study, coevolution of *C. elegans *with a bacterial pathogen selected for higher outcrossing rates in mixed mating experimental populations [28]. Direct experimental support for a change in recombination rate compatible with the Red Queen Hypothesis in obligatory outcrossing species has so far only been reported in one of our earlier studies using *Tribolium castaneum *as the host and *Nosema whitei *as its parasite [29]. This finding could not be confirmed in a follow-up study [30], yet post-hoc checks suggested that the extant genetic variation in the hosts probably was too small to sustain an adaptive response (unpubl. data).

The *Tribolium-Nosema *system is an ideal test ground for Red Queen-related questions, since it meets the key assumptions of the hypothesis [31-34]. For example, *N. whitei *is an obligately killing parasite [31], which means that there is a severe fitness cost for parasites that are not able to infect or kill their host. In fact, strong selection on the parasite can favour higher recombination rates in the host, even if selection on the host is weak [10]. Furthermore, there is a substantial epistatic component of resistance of *T. castaneum *to infection with *N. whitei *[32,33]. Epistasis in turn, generates linkage disequilibrium, which can be broken down by recombination, thereby creating fitter genotypes. Finally, host genotype versus parasite genotype interactions are found for *N. whitei *interacting with *T. castaneum *[34].

Here, we compared - relative to uninfected control lines - the recombination rates in four replicate *T. castaneum *lines that were allowed to coevolve with a mixture of eight *N. whitei *isolates. More specifically, recombination frequencies were measured in males from both treatments after eleven discrete generations, using microsatellite markers that bordered ten intervals on the genome, which were distributed over four linkage groups. Using this method enabled us to measure recombination directly in the individuals under selection, as opposed to earlier studies that measured recombination in the offspring of the selected individuals, and provided us with a better genomic coverage than in any previous study [29,30]. The experimental design was such that control (no parasite) and coevolution (with parasites) treatments were paired within each of the eight replicate lines of the host. We chose the conditions of the experiment (number of generations, replicate lines, etc.) based on the experience and results gained in previous experiments with the same study system [29,30].

Earlier findings from the same coevolution experiment showed that coevolved host lines maintained higher genetic diversity than control host lines [14]. If genetic diversity in the coevolved lines is maintained because of negative frequency-dependent selection by the parasite, it could be assumed that in every generation rare host genotypes created by recombination enjoy an advantage. When parasite-mediated selection is strong enough, one would thus expect to see higher recombination frequencies evolve in the coevolving beetle lines. Such an increase in recombination frequencies, in combination with the observed maintenance of genetic diversity, can therefore be seen as evidence for the Red Queen Hypothesis in an obligatory outcrossing species. Here, we show that host recombination did indeed increase during coevolution with a parasite, and that this change is compatible with fluctuating selection exerted by the parasite.

## Results and discussion

**Figure 1:**
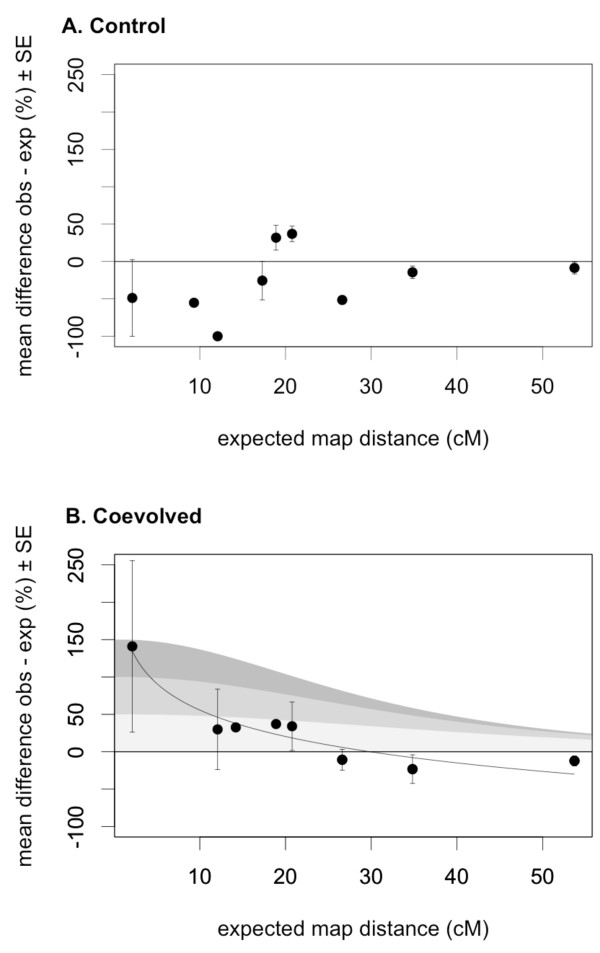
**Mean relative difference between observed and expected map distance against the expected map distance**. The figure shows all data, i.e. all recombination measurements in all intervals of all lines (35 distinct recombination measurements in control lines, and 24 distinct recombination measurements in coevolved lines). Recombination frequencies were transformed into map distances. Map distances were averaged over interval and line. **a**, The relative difference does not deviate from a non-difference line and does not correlate with interval length (linear regression, n = 9, F_1, 7 _= 0.986, P = 0.354), suggesting that recombination in the control treatment did not deviate from expected recombination. **b**, In the coevolving lines a significant logarithmic relationship was observed (linear regression, n = 8, F_1, 6 _= 64.310, P < 0.001, R^2 ^= 0.915), which might indicate an overall increase in recombination. The grey areas represent the theoretical change in observed recombination rate, in terms of percentage, in the cases of a 50% (light grey), 100% (medium grey) and a 150% (dark grey) genome-wide increase in actual map distance. Kosambi's map function was used to convert map distances into recombination frequencies. The areas illustrate that for large intervals it might be hard to detect a change in recombination frequency, even in the case of a substantial increase in the actual map distance.

After 11 generations of experimental coevolution, the mean difference between observed and expected Kosambi's map distance in the control lines (without parasites) did not relate to the expected map distance and did not significantly deviate from the line of no difference (Figure 1a). By contrast, there was a significant logarithmic relationship between the mean difference of observed and expected map distance, and the length of the genomic interval (map distance) in the coevolved lines (Figure 1b). In addition, the slope of the coevolution regression line was significantly different from the slope of the control regression line (linear regression, β3 = 69.212, t_13 _= 3.796, P = 0.002). Although it appears, at first sight, that only short intervals increased in recombination frequency in the coevolution treatment, the longer intervals (right hand side) might still be reconciled with an overall increase in recombination. If the true recombination frequency has increased at the same rate all over the genome, across all intervals, then it will be harder to observe this increase in larger intervals (as illustrated in Figure 1b). In those relatively large intervals, the observed recombination rate is closer to its maximum of 50%, and therefore a similar increase in actual recombination frequency results in a relatively small increase in observed recombination values. Even though we used Kosambi's map function to convert observed recombination values to map distances, any such analysis still suffers from this issue, and changes in the real recombination rate in larger intervals are likely to remain undetected. Furthermore, negative values at larger intervals for both treatments (Figure 1a, b) suggest that there was a systematic bias towards lower recombination rates in the lines used here relative to the expected map distances. This might simply be due to the fact that the standard genetic map of *T. castaneum *[35], from which the expected map distances were derived, was based on different beetle populations than the ones used in this experiment. Presumably, the real "line of no difference" of our data lies below the zero lines in the graphs.

**Figure 2:**
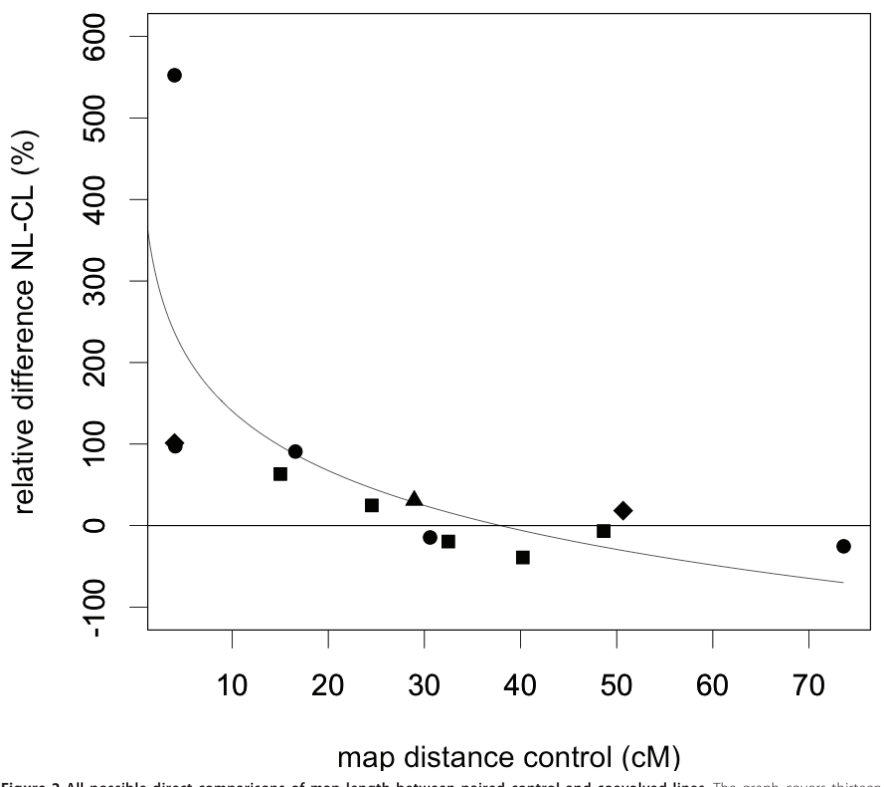
**All possible direct comparisons of map length between paired control and coevolved lines**. The graph covers thirteen corresponding (paired) intervals (x-axis). To account for zero values, one observation was added to all recombinant counts and to all total counts; hence, recombination frequency is calculated as: n_recombinants _+ 1/n_total _+ 1. Recombination frequencies were transformed into map distances. Shown is the significant logarithmic relationship between the relative difference in percentages of coevolved (NL) and control lines (CL), and the observed map lengths in the control lines (linear regression, n = 13, F_1, 11 _= 10.188, P = 0.009, R^2 ^= 0.481). This indicates that observed recombination frequencies are higher in coevolved lines compared to control lines. Symbols indicate from which line each data point originates: a triangle for line 3, a square for line 4, a circle for line 5, a diamond for line 6.

Thirteen direct comparisons could be made between the map distance of the same interval for beetle lines that were paired over control and coevolved treatments (i.e. the same lines having been split and assigned to the two treatments). The direct comparison showed essentially the same pattern as described above, since for the short intervals map distances were higher in the coevolved lines than in the control lines. This difference disappeared with increasing interval size (Figure 2), something that can again be explained by the fact that changes in observed recombination frequency in larger intervals are relatively small and therefore harder to detect. The observed pattern might thus indicate an overall increased recombination frequency.

Showing that host recombination has increased during coevolution with a parasite does not provide unequivocal evidence for the Red Queen Hypothesis. For example directional selection, in combination with the effects of limited population size [36], might alternatively contribute to this result. However, directional selection for insecticide resistance did not result in higher recombination frequencies in this system [29]. Instead, there are several findings suggesting that our result is tied to the process of antagonistic coevolution. For example, coevolving populations maintained higher levels of heterozygosity and allelic diversity than control populations in this experiment [14], which is another prediction based on the Red Queen Hypothesis, and which does not match the scenario of directional selection. At the same time we could show that higher levels of heterozygosity is not *per se *the reason for higher parasite resistance in *T. castaneum*, as inbreeding does not increase the overall susceptibility of the beetles to parasitic infections (for both our case of *N. whitei *(Kerstes et al., in prep) as well as for infections by the rat tapeworm *Hymenolepis diminuta *[37]). In a similar fashion, there was no significant difference in F_IS_-values (inbreeding coefficient) between coevolved and control populations in this experiment [14], and outcrossed beetles were not more resistant to *N. whitei *than their most resistant parent in general ([32], Kerstes et al., in prep). Furthermore, parasite-induced mortality was shown to fluctuate during the first generations of the coevolution experiment [38]. All these findings combined suggest that genetic diversity is maintained in the host populations not because of heterozygote advantage/overdominance, but likely as a result of fluctuating selection exerted by the parasite.

Since the current stock host populations differ from those used in the earlier experiments that produced comparable patterns [29], it seems highly unlikely that our current results are caused by the presence of a particularly favourable initial linkage disequilibrium between a recombination modifier and a locus under selection from the parasites [29]. Furthermore, the replicate populations were all started from different initial linkage disequilibria. It appears that recombination frequency in populations of *T. castaneum *responds very quickly to selection [39], which suggests that our result is due to actual, adaptive evolutionary change. Indeed, no evidence was found for an infection-induced plastic change in recombination in this system [30].

It is nevertheless conceivable that prolonged exposure to non-coevolving parasites would lead to an increase in host recombination frequencies, too. However, in a previous study with the same experimental paradigm we found that populations of *T. castaneum *that were exposed to randomly selected, non-evolving, parasites for 12 generations did not differ in recombination rate from controls [29]. Hence, even though we cannot forcefully rule out an effect of simple exposure to parasites in a single all-encompassing experiment, and since there are potentially many other alternative explanations besides fluctuating selection due to the Red Queen process (e.g. genetic correlations between resistance and other traits [40]) to explain the maintenance of genetic diversity and the increase in recombination rate, currently the Red Queen Hypothesis remains the major contender.

We also did observe that host populations became more resistant during coevolution with the parasite [41], contrary to a scenario of pure negative frequency-dependent selection where no such mean change is expected [3]. Host resistance to infection with *N. whitei *has been shown to be a complex trait [32], and one could imagine that some components of resistance can be under fluctuating selection, while others are under directional selection. The idea that, in particular during the early phase of the experiment, fluctuating and directional selection were both important fits the observation that parasite-induced mortality was shown to fluctuate during the first generations of the coevolution experiment, while there was a general decrease of parasite-induced mortality (and infectivity) over time, and fluctuations only disappeared in the longer term [38].

The observed decrease in parasite infectivity might indicate that the hosts are ahead in the arms race with their parasites, because a loss in infectivity can only be considered maladaptive for the parasite. We interpret this finding as a possible result of more rapid depletion of genetic variation in the parasite populations than in their coevolving antagonists. As our experimental conditions did not allow for migration between replicate populations, and because *N. whitei *is asexual [42], the parasite population would indeed lose variation that might not be compensated by mutations. At the same time, under our experimental conditions, the benefit of increased recombination frequencies for the host population might disappear in the long term for the same reason.

## Conclusions

We found that, after 11 generations of coevolution, recombination frequency in the host population was increased. Based on insights into genotypic [14] and phenotypic [38] changes that occurred during the coevolution experiment, the observed increase in host recombination frequency is likely to be the result of fluctuating selection exerted by the parasite during early stages of coevolution, although we cannot completely exclude alternative explanations. In this study we benefited from a better genomic coverage, direct recombination measurements, and an improved understanding of the mechanisms behind our observations. We can show that host-parasite coevolution affects the evolution of recombination. Together with the findings of other recent studies [15,28,43], we provide experimental support for the Red Queen Hypothesis as a theory to explain the evolution and maintenance of recombination.

## Methods

### Host-parasite coevolution

All beetles were maintained at 32°C, 70% humidity, in 24 h darkness, on standard medium (type 550 'Knospe' organic flour containing 5% dried yeast). All beetle stock populations used in the experiment were kept at large population sizes (> 200) in stable, parasite-free environments for at least 50 generations prior to the coevolution experiment. Eight experimental beetle lines were set up, each as a unique combination of two stock populations (seven stock populations were used in total). Fifty virgin females from one stock line were crossed with fifty virgin males from the other, the reciprocal crosses were made with equal numbers, and all offspring were pooled to serve as the starter generation of the experimental line. We chose to set up our experimental lines in such a way because stock populations are likely to harbour reduced genetic diversity.

Subsequently, each line was split up in two treatments: coevolution and control. All eight lines were thus represented in both treatments. Corresponding lines (the same line in both the control and the coevolution treatment) were considered paired in the analyses, and consequently each given pair has the same genetic background. The lines in the coevolution treatment were subjected to selection by coevolving *N. whitei*, while the same lines in the control treatment were always kept and handled in identical ways, except that the medium was parasite-free and the hosts thus uninfected. *N. whitei *is a directly transmitted microsporidian parasite that is - from all what is known so far - reproducing asexually [42]. Every generation 500 unsexed beetles from the previous generation were used as breeders to initiate the next host generation. A mixture of eight different *N. whitei *isolates (to ensure sufficient standing genetic variation in the parasite population) was used to infect the first generation of beetles in the coevolution treatment, by mixing spores into the standard medium at a concentration of 2 * 10^4 ^spores g^-1 ^of medium. Every generation dead larvae harbouring *N. whitei *spores were collected, and a *N. whitei *powder was created by grounding and sieving the larvae. This was done for each experimental line separately. The next generation of each coevolving beetle line was then infected with their own unique mix of *N. whitei *spores derived from dead beetle larvae from the generation before. By selecting parasites that were able to infect and kill their hosts, and hosts that were able to survive the previous generation, it was ensured that both coevolving partners were exerting antagonistic selective pressures on each other [41]. Recombination frequency was estimated from beetles taken from the eleventh generation of the experiment.

### Recombination measurement

Ten males from each line in both treatments were collected as pupae. Each reproductively mature male that emerged from these pupae was then crossed with one virgin female from an unrelated marker strain (strain LG1). Six (from the eight) experimental lines were chosen based on the success of the crosses, and eight males per line (from the ten that were crossed with a LG1 female) were selected in both the control and the coevolved treatment. These beetles were scored for heterozygosity of 11 microsatellite markers (Table 1), distributed over four linkage groups (nrs. 3, 6, 7 and 10), using published methods [14].

**Table 1:** Primer characteristics of the used microsatellite markers (* derived from [44])

*Primer name*	*Linkage group*	*Position*	*Repeat*	*Forward primer*	*Reverse primer*
Tca-3.19*	3	5940315	AAT	CCATTGCAGATTGTAGGGTGT	GTTTTTACAGCGCCGAACAT
LGIII2	3	6593045	AAT	CATCACTTGGGTGCTTTATCC	CAATACCTGAATGTGTGTGTGC
LGIII3	3	9169070	ATA	CACTATTTCCGCATATTGTTGC	TTATCCCTCTTTGGCAGACG
LGVI	6	9124774	TAA	CAAAGCACTCATGTACGAAACC	CCTCTTATTGACTTGTGTTATGACC
Tca-6.11*	6	5965031	AAT	TAGTCTGCCGGCTGGTAAGT	AGCGACCGACATTTGTGTTT
Tca-6.2*	6	3982475	A	TTTTTGTTGGGACACCCTGTA	TTGCGACGTATTTTCATTCG
Tca-7.2*	7	4598114	A	GCTCGATTGGTAGGTGTGGT	AAAGCCTTTCACCTCCATTCT
LGVII1	7	904671	AAT	TTGTCTCTTTCAGGCCAAGG	GCTGAAATACTGGTCTGAGATGC
LGVII2	7	5833915	ATT	AAGGCATGCTTTGGTTCC	TGAATGCCGAAGACTAGTATGG
Tca-10.1*	10	4582080	AAT	AAATTCTCGGCTTTTTGGGT	GAGCTGGCGGTTATATTGGA
LGX4	10	8139860	CGG	ATAGTTGCGCGCCTTTCG	ACATCACTGCGTCATGCTAGG

For the molecular analyses, beetles from four control lines and their corresponding, paired coevolved lines were chosen. So in the end, only four of the eight experimental lines were used to score recombination. Initial choice of experimental lines was based on the availability (i.e. homozygous loci cannot reveal recombination events) and distribution (i.e. for a genome-wide coverage) of heterozygous loci within individuals. Hence, each line could have a different combination of diagnostic loci that border intervals of varying lengths. From each selected line, three males (from the eight that were scored for heterozygosity) were selected based on the same criteria of availability and distribution of loci, and up to 24 offspring per male were genotyped with the appropriate markers. Recombination rate was calculated as the number of recombinant offspring divided by the total number of screened offspring. A total of 576 offspring were genotyped to score recombination in 24 males (four lines, two treatments, three males per treatment per line), and recombination was successfully scored in 1'416 cases (meaning that recombination was scored in an average of about 2.5 interval per male).

The observed recombination frequency does not necessarily reflect the true recombination frequency, especially in larger genomic intervals [45]. Multiple numbers of recombination events will downward bias our estimate, as they cancel each other out and can result in what appears to be a non-recombinant genotype [46]. Besides double recombination events, also crossover interference could affect the observed recombination frequency. As crossover interference has been shown to occur in *T. castaneum *[30], we decided to use Kosambi's map function [47] to calculate map distances from our observed recombination frequencies. Kosambi's function corrects for both double recombination events and the occurrence of crossover interference [46].

### Expected map distance

For each linkage group we acquired the genetic position (cM) and sequences of BAC (Bacterial Artificial Chromosome) ends and ESTs (Expressed Sequence Tag) that were used to create a genetic linkage map of *T. castaneum *[35]. We performed a BLAST (Basic Local Alignment Search Tool) search of the sequences against the *T. castaneum *genome [48] to get their physical position (bp). Per linkage group, genetic position was plotted as a function of physical position, and a fourth order polynomial was fitted through the points (SPSS 19 for Mac OS X; all functions explained more than 98% of the variation). Knowing the physical position of the two markers bordering a given interval, we were able to calculate an expected map distance for each interval [23].

All experiments have been done in accordance with the regulations of ETH and Switzerland.

### Data analysis

Linear regression (SPSS 19 for Mac OS X) was used to investigate several possible relationships. In both cases where we found a significant relationship, logarithmic trend lines (y = intercept + slope * ln(x)) provided a better fit than straight trend lines (y = intercept + slope * x).

To be able to compare the slopes of the regression lines for the relationship between the mean relative difference between observed and expected map distance and the expected map distance of the control and the coevolution treatment (Figure 1), all expected map distances were ln-transformed. The mean relative differences for both treatments were combined in one dataset, and the variables were defined as follows: y contains the difference values, x1 is a dummy variable to split up the data set in the two treatments (0 = coevolved, 1 = control), ×2 contains the ln-transformed expected map distances, and ×3 is the product of x1 and ×2. Then a linear regression was performed using the following model: y = β0 + β1*×1 + β2*×2 + β3*×3. In this model β3 represents the difference between the slopes of the regression lines of the two different treatments. It was tested if β3 deviates significantly from zero.

## Authors' contributions

NAGK carried out the recombination measurements, analysed the data, and drafted the manuscript. CB carried out the coevolution experiment. PS and KMW conceived the study and coordinated it. All authors participated in the design of the study, read and contributed to the manuscript, and approve the final manuscript.
